# Hyperelastic Microcantilever AFM: Efficient Detection Mechanism Based on Principal Parametric Resonance

**DOI:** 10.3390/nano12152598

**Published:** 2022-07-28

**Authors:** Amin Alibakhshi, Sasan Rahmanian, Shahriar Dastjerdi, Mohammad Malikan, Behrouz Karami, Bekir Akgöz, Ömer Civalek

**Affiliations:** 1 Department of Mechanical Engineering, Science and Research Branch, Islamic Azad University, Tehran 1477893855, Iran; alibakhshiamin@yahoo.com; 2 Department of Systems Design Engineering, University of Waterloo, Waterloo, ON N2L 3G1, Canada; s223rahm@uwaterloo.ca; 3 Division of Mechanics, Civil Engineering Department, Akdeniz University, Antalya 07058, Turkey; dastjerdi_shahriar@yahoo.com (S.D.); bekirakgoz@akdeniz.edu.tr (B.A.); civalek@yahoo.com (Ö.C.); 4 Department of Mechanics of Materials and Structures, Faculty of Civil and Environmental Engineering, Gdańsk University of Technology, Gabriela Narutowicza 11/12, 80-233 Gdańsk, Poland; 5 Department of Mechanical Engineering, Marvdasht Branch, Islamic Azad University, Marvdasht 73711-13119, Iran; behrouz.karami@miau.ac.ir; 6 Department of Medical Research, China Medical University Hospital, China Medical University, Taichung 406, Taiwan

**Keywords:** atomic force microscopy, hyperelastic microcantilever, softening resonance, non-contact cantilever, shooting and arc-length continuation method, developed Galerkin method

## Abstract

The impetus of writing this paper is to propose an efficient detection mechanism to scan the surface profile of a micro-sample using cantilever-based atomic force microscopy (AFM), operating in non-contact mode. In order to implement this scheme, the principal parametric resonance characteristics of the resonator are employed, benefiting from the bifurcation-based sensing mechanism. It is assumed that the microcantilever is made from a hyperelastic material, providing large deformation under small excitation amplitude. A nonlinear strain energy function is proposed to capture the elastic energy stored in the flexible component of the device. The tip–sample interaction is modeled based on the van der Waals non-contact force. The nonlinear equation governing the AFM’s dynamics is established using the extended Hamilton’s principle, obeying the Euler–Bernoulli beam theory. As a result, the vibration behavior of the system is introduced by a nonlinear equation having a time-dependent boundary condition. To capture the steady-state numerical response of the system, a developed Galerkin method is utilized to discretize the partial differential equation to a set of nonlinear ordinary differential equations (ODE) that are solved by the combination of shooting and arc-length continuation method. The output reveals that while the resonator is set to be operating near twice the fundamental natural frequency, the response amplitude undergoes a significant drop to the trivial stable branch as the sample’s profile experiences depression in the order of the picometer. According to the performed sensitivity analysis, the proposed working principle based on principal parametric resonance is recommended to design AFMs with ultra-high detection resolution for surface profile scanning.

## 1. Introduction

Atomic force microscopy (AFM) is a device used to study materials’ properties and surface structure in micro- and nanometer dimensions [1,2,3]. Flexibility, multiple potential signals, and the ability to operate the device in different modes have enabled researchers to study AFMs at different levels and under different environmental conditions. This device makes it possible to examine conductive or insulating surfaces, soft or hard, cohesive or powdery, biological and organic or inorganic. The general architecture of an AFM includes a microcantilever with a sharp tip at the free end contacting with the surface of samples [4]. The AFMs are applicable devices that have been widely used in so many scientific purposes, such as chemistry [5], materials science [6], medical and biophysics [7], and biological and colloidal applications [8].

Such as many structures that are influenced by the time [9,10,11,12], the operation of AFMs may be time-dependent and, consequently, under dynamic conditions. Thus, most previous studies in this field have focused on the dynamic performance of AFMs. The chaotic motion and bifurcation of a tapping mode AFM were examined by Yagasaki [13]. Bahrami and Nayfeh [14] explored the nonlinear oscillation of a tapping mode AFM by using a high-dimensional Galerkin discretization technique and a four-step Adams–Moulton method. They modeled the microcantilever based on the Euler–Bernoulli beam theory. The flexural and torsional vibrations of an AFM were investigated by Kahrobaiyan et al. [15], who performed a sensitivity analysis. Arafat and co-investigators [16] analyzed the frequency–amplitude response of a contact mode AFM analytically using the multiple time-scale method. Dastjerdi and Abbasi [17] studied the free vibration of a cracked AFM, incorporating the influence of crack size and its location. They utilized the transfer matrix method to solve the cracked system. Also, they concluded that existing a crack (with a specific size and location) on the AFM cantilever can be beneficial for controlling some unwanted phenomena. Mahmoudi et al. [18] studied the resonance of a non-contact AFM using harmonic balance and multiple time-scale methods. Saeidi et al. [19] explored forced vibrations of an AFM, taking the temperature and contact effects into account. Ahmadi and co-workers [20] studied free and forced oscillations of AFM with rectangular and V-shaped and employed the finite element analysis. Kouchaksaraei and Bahrami [21] proposed a new multifrequency excitation for AFM in a non-contact regime. They analyzed the sensitivity to the Hamaker constant and the initial tip–sample distance to enhance the performance of the AFM. In most previous studies investigating dynamic characteristics of AFMs, the structural materials have been a linear type. This means that the linear elasticity governs their behavior with small strain and deformation.

There has been an increasing interest in soft and hyperelastic materials in recent decades, because they are lightweight, cheap, compatible with many flexible structures, and show ease of fabrication. The relation between the strain and stress for hyperelastic materials is nonlinear. Besides, the strain in hyperelastic materials is moderate or large, and they undergo large deformation. These are the main differences between hyperelastic materials and linear elastic materials. Hyperelastic materials are structural materials of many electromechanical systems such as actuators, sensors, and energy harvesters. More specifically, hyperelastic materials have been widely used for electro-active-based systems [22,23,24,25]. Up to now, hyperelastic materials have been designed in different geometries, e.g., beams [26,27,28,29], plates [30,31], and shells [32,33]. Reviewing the previous studies shows a growing body of literature on using hyperelastic materials in different systems. Due to these advantages of hyperelastic materials and their broad applications, this paper proposes an AFM made of such materials, unique in simulation and application. Most recently, the dielectric elastomer actuators have been proposed [34] for AFM, and because these kinds of actuators are hyperelastic, exploring hyperelastic-based AFM may be of significant importance.

As is reported in the literature review, it can be seen that there is a lack of nonlinear study on vibration analysis of AFM cantilevers made of hyperelastic material. Consequently, thorough research on nonlinear frequency analysis of AFM cantilevers has been conducted based on the hyperelasticity approach. This paper is structured as follows. Section 2 derives the equation of motion based on a mathematical model containing material and geometrical nonlinearities, size effects, and finite rotations. In Section 3, the governing equation is discretized with a developed Galerkin decomposition method and solved through the shooting method combined with the arc-length continuation method. Finally, in Section 4, numerical results have been provided to study different dynamical behaviors of the AFM cantilever under the assumed conditions.

## 2. Mathematical Modeling

**Figure 1:**
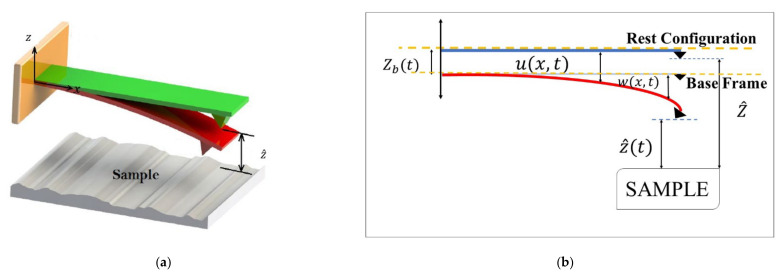
The schematic view of an AFM’s cantilever scanning a sample. (**a**) 3D view of the AFM. (**b**) 2D view of the cantilever.

Figure 1 demonstrates the schematic view of an AFM cantilever simulated in this paper. The system consists of a hyperelastic microcantilever with a sharp tip at the free end. A Cartesian coordinate system (x,y,z) is employed to define the configuration of the AFM. The base vectors in x,y,z−directions are $e_{1}$, $e_{2}$, and $e_{3}$, respectively. The microcantilever’s length, thickness, and width are denoted via L, d, and b, respectively.

Figure 1a is a 3D schematic and perhaps does not provide more details. On the other hand, Figure 1b gives more details. The AFM consists of a microcantilever mounting on a base with piezoelectric patches as the excitation source (see Figure 1b). As depicted in Figure 1b, $\hat{Z}$ is the distance between the tip and the sample when the microbeam is located at rest, and $w\left(x,t\right)$ is the transverse displacement of the microcantilever with respect to the base frame. The transverse base excitation is $Z_{b}\left(t\right)=d_{0}\sin\left(\omega t\right)$, in which $d_{0}$ and ω are the amplitude and frequency, respectively. The total deflection is $u\left(x,t\right)=w\left(x,t\right)+Z_{b}\left(t\right)$. Regarding these parameters, the instantaneous tip–sample separation is indicated by $\hat{z}\left(t\right)=\hat{Z}-w\left(L,t\right)-Z_{b}\left(t\right)$.

The governing equations are derived under the following assumptions: (1) the effect of rotary inertia and shear deformation is disregarded, i.e., the microbeam is modeled based on the Euler-Bernoulli beam theory. (2) both geometric and material nonlinearities are considered. (3) the rotation of the microcantilever is considered to be a moderate type, which means that ∂w/∂x is moderate. In other words, the rotation is of order $\sqrt{\epsilon }$, in which ϵ≪1 stands for a small parameter (for more details on deriving moderate rotation see [35]). (4) the size effect is considered using the modified couple stress theory. (5) the axial displacement is neglected.

**Figure 2:**
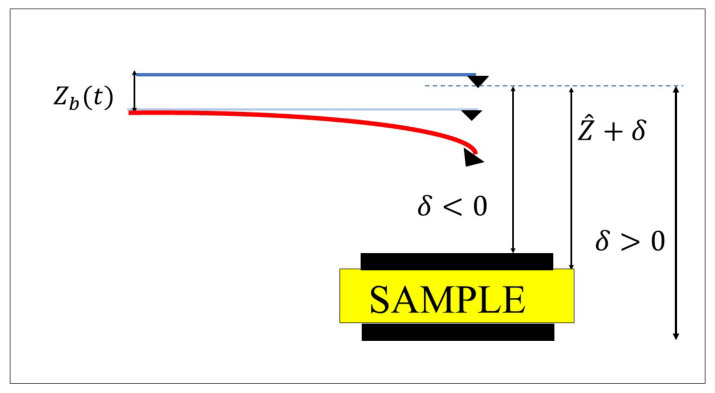
Schematic representation of the increase and decrease in surface profile.

In reality, a sample subjected to probing may have an inhomogeneous surface, thus leading to surface depression or an increase in surface height. During probing, these increases in surface height may affect the performance of the hyperelastic AFM. As shown in Figure 2, the location of surface depression and increase in surface height assumed in the system are shown. In Figure 2, δ refers to any variation in the height of the sample profile with respect to the baseline, i.e., $\hat{Z}+\delta =\hat{Z}+0=\hat{Z}$.

### 2.1. Beam Theory with Finite Rotation and Deformation

The components of the displacement vector $u=u_{1}{\hat{e}}_{1}+u_{2}{\hat{e}}_{2}+u_{3}{\hat{e}}_{3}$ can be given as [36].

(1) $$\left\{\begin{array}{ll} u_{1}=z\theta_{x}, & \;\;u_{2}=0\\ u_{3}=w\left(x,t\right)\\ \theta_{x}\equiv -\frac{\partial w}{\partial x} \end{array}\right.$$

In Equation (1), $\theta_{x}$ is a defined parameter, namely, the minus of the slope of the transverse displacement.

The strain–displacement relations for the system are obtained according to the finite deformation. To this end, the von Kármán theory originated from Green’s strain theory is used as follows [37]

(2) $$\left\{\begin{array}{l} E_{xx}=\varepsilon_{xx}{}^{\left(0\right)}+z\varepsilon_{xx}{}^{\left(1\right)}\\ E_{zz}=\varepsilon_{zz}{}^{\left(0\right)} \end{array}\right.$$

in which

(3) $$\left\{\begin{array}{l} \varepsilon_{xx}{}^{\left(0\right)}=\frac{1}{2}{\left(\frac{\partial w}{\partial x}\right)}^{2}\\ \varepsilon_{xx}{}^{\left(1\right)}=\frac{\partial \theta_{x}}{\partial x}\\ \varepsilon_{zz}{}^{\left(0\right)}=\frac{1}{2}\theta_{x}{}^{2} \end{array}\right.$$

In Equation (2), $E_{xx}$ is the strain in the x-direction, and $E_{zz}$ stands for the strain in the z-direction.

The potential energy is expressed in terms of the strain energy function for hyperelastic materials. The strain energy can be formulated based on the displacement as mentioned above and strain components incorporating the finite deformation, finite rotation, and size effect as follows [38,39].

(4) $$\Psi =\frac{1}{2}\left(a_{1}E_{xx}^{2}+a_{2}E_{zz}^{2}+a_{3}{\left(\theta_{x}{}^{’}\right)}^{2}+2a_{4}E_{xx}E_{zz}\right)$$

in which

(5) $$\left\{\begin{array}{l} a_{1}=a_{2}=2\mu +\lambda \\ a_{4}=\lambda \\ a_{3}=2\mu \ell^{2} \end{array}\right.$$

In Equation (5), ℓ is the internal length-scale parameter capturing the size effect, which is defined as the square root of the ratio of the moduli of curvature to the shear [38,40] and is generally quantified as one-half or one-fourth of the thickness of the structure in theoretical analyses. It is noted that the value of this parameter from experimental evidence for hyperelastic microstructures seems to be rare. For more details on experimental methods of calculating l, you may refer to [41].

Moreover, in Equation (15), $\mu =E/2\left(1+\nu \right)$ stands for the shear modulus, and $\lambda =\left[E\nu \right]/\left[\left(1+\nu \right)\left(1-2\nu \right)\right]$ is the Lamé’s constant (ν is the Poisson’s ratio). It is mentioned that Equation (5) has shown its applicability for hyperelastic structures in previous literature.

The hyperelastic potential energy is therefore calculated as:

(6) $$U_{el}=\int_{\bar{V}}\Psi d\bar{V}$$

where $\bar{V}$ is the volume of the microcantilever.

Substituting Equation (2) into Equation (4), then inserting it into Equation (6), the final form of the hyperelastic energy is presented as:

(7) $$U_{el}=\int_{0}^{L}\left[\frac{1}{8}a_{1}A{\left(\frac{\partial w}{\partial x}\right)}^{4}+\frac{1}{2}a_{1}I{\left(\frac{\partial^{2}w}{\partial x^{2}}\right)}^{2}+\frac{1}{8}a_{2}A{\left(\frac{\partial w}{\partial x}\right)}^{4}+\frac{1}{2}a_{3}A{\left(\frac{\partial^{2}w}{\partial x^{2}}\right)}^{2}+\frac{1}{4}a_{4}A{\left(\frac{\partial w}{\partial x}\right)}^{4}\right]dx$$

With the cross-section area A=bd and the second moment of area $I=bd^{3}/12$.

### 2.2. Tip–Sample Interaction

At the free end of the microcantilever, there is the interaction force between the tip and sample. In the present paper, as a test case, a non-contact tip–sample interaction so-called the van der Waals non-contact force is applied, i.e., [14],

(8) $$F_{\mathrm{vdW}}=-\frac{HR}{6{\hat{z}}^{2}}$$

in which $F_{\mathrm{vdW}}$ stands for the van der Waals non-contact force, H is the Hamaker constant, and R is the radius of the spherical tip apex.

The system’s potential energy due to the tip–sample interaction is $V_{\mathrm{tp}}=-\int F_{\mathrm{vdW}}\;d\hat{z}$. By calculating this integration, the final form of the tip–sample interaction potential energy for the non-contact region will be formulated as [14]:

(9) $$U_{\mathrm{vdW}}=-\frac{HR}{6\left[\hat{Z}-w\left(L,t\right)-Z_{b}\left(t\right)\right]}$$

### 2.3. Kinetic Energy

The total kinetic energy of the AFM cantilever can be expressed below (ρ is the mass density of the microcantilever).

(10) $$U_{k}=\frac{1}{2}\int_{0}^{L}\rho A{\left[\left(\frac{\partial w}{\partial t}\right)+\left(\frac{\partial Z_{b}\left(t\right)}{\partial t}\right)\right]}^{2}dx$$

### 2.4. Surrounding Damping Force

The constitutive material of the AFM is hyperelastic, for example, it is rubbery or elastomeric. For such materials, viscoelasticity plays a major role that should be considered in the analysis. In this paper, a linear damping model is assumed, while other complicated types of damping can also be used.

The amount of work created by the viscous surrounding medium is formulated as ($c_{d}$ is the viscous damping coefficient).

(11) $$W_{D}=-c_{d}\int_{0}^{L}\left[\left(\frac{\partial w}{\partial t}\right)+\left(\frac{\partial Z_{b}\left(t\right)}{\partial t}\right)\right]wdx$$

### 2.5. Hamilton’s Principle and Equation of Motion

The governing equation can be easily derived using variational approaches by obtaining the energies and works that appear in the system [42,43,44]. The partial differential equation governing the motion of the AFM is derived using the following Hamilton’s principle as one of the variational approaches:

(12) $$\int_{t_{1}}^{t_{2}}\left[U_{k}-U_{s}\right]dt+\int_{t_{1}}^{t_{2}}\delta W_{D}dt=0$$

where $U_{s}$ shows the total potential energy of the system, which for the non-contact region is: $U_{s}=U_{el}+U_{\mathrm{vdW}}$.

Eventually, substituting the expression of the kinetic and potential energies and the work of damping force into Hamilton’s principle gives the following formulation:

(13) $$\begin{array}{cc} \rho A\frac{\partial^{2}w}{\partial t^{2}}+\rho A\; & \frac{\partial^{2}Z_{b}\left(t\right)}{\partial t^{2}}+c_{d}\frac{\partial w}{\partial t}+c_{d}\frac{\partial Z_{b}\left(t\right)}{\partial t}-\frac{3}{2}a_{1}A\left(\frac{\partial^{2}w}{\partial x^{2}}\right){\left(\frac{\partial w}{\partial x}\right)}^{2}\\ & +a_{1}I\left(\frac{\partial^{4}w}{\partial x^{4}}\right)-\frac{3}{2}a_{2}A\left(\frac{\partial^{2}w}{\partial x^{2}}\right){\left(\frac{\partial w}{\partial x}\right)}^{2}+a_{3}A\left(\frac{\partial^{4}w}{\partial x^{4}}\right)\\ & -3a_{4}A\left(\frac{\partial^{2}w}{\partial x^{2}}\right){\left(\frac{\partial w}{\partial x}\right)}^{2}=0 \end{array}$$

It is noteworthy that the mathematical definition of the boundary conditions is obtained from Hamilton’s principle. Consequently, the boundary condition definition can be formulated as the following equations.

(14) $$\left\{\begin{array}{l} w\left(0,t\right)=\frac{\partial w}{\partial x}\left(0,t\right)=0\\ \;\;\;\;\;\;\;\frac{\partial^{2}w}{\partial x^{2}}\left(L,t\right)=0\\ a_{1}I\frac{\partial^{3}w}{\partial x^{3}}\left(L,t\right)+a_{3}A\frac{\partial^{3}w}{\partial x^{3}}\left(L,t\right)=-\frac{HR}{6{\left[\hat{Z}-w\left(L,t\right)-\hat{y}\left(t\right)\right]}^{2}} \end{array}\right.$$

The boundary conditions for cantilevers state that the deflection and slope at the point x=0 (at the fixed end) are equal to zero, and the bending moment and shear force at x=L (at the free end) are equal to zero. From Equation (14), it is observed that the difference between the boundary conditions of the cantilever and AFM appears in the shear force in such a way that for the AFM it is not equal to zero any longer and is nonhomogeneous and time-dependent due to tip–sample interaction.

### 2.6. Nondimensionalization

In this section, the equation of motion and boundary conditions are made dimensionless. Hence, the dimensionless parameters can be presented as follows [45]:

(15) $$x^{*}=\frac{x}{L},\;w^{*}=\frac{w}{\hat{Z}},{\bar{d}}_{0}=\frac{d_{0}}{\hat{Z}},\;t^{*}=t\sqrt{\frac{EI}{\rho AL^{4}}},c=\frac{c_{d}L^{4}}{EI}\sqrt{\frac{EI}{\rho AL^{4}}},\Omega =\omega \sqrt{\frac{\rho AL^{4}}{EI}}\;d_{1}=\frac{a_{1}I}{EI},d_{2}=\frac{a_{3}A}{EI}=\frac{2\mu Al^{2}}{EI},d_{3}=\frac{3a_{1}A{\hat{Z}}^{2}}{2EI},d_{4}=\frac{3a_{2}A{\hat{Z}}^{2}}{2EI},d_{5}=\frac{3a_{4}A{\hat{Z}}^{2}}{EI}\;\bar{Z}=\frac{\hat{Z}}{\hat{Z}},\;d_{6}=\frac{HRL^{3}}{6EI{\hat{Z}}^{3}}$$

The non-dimensional form of the equation of motion and boundary conditions are obtained as (the asterisk notation is disregarded for simplification):

(16) $$\frac{\partial^{2}w}{\partial t^{2}}+c\frac{\partial w}{\partial t}-{\bar{d}}_{0}{\;\Omega }^{2}\sin\left(\Omega t\right)+c{\bar{d}}_{0}\;\Omega \cos\left(\Omega t\right)+\underset{\alpha }{\underbrace{\left(d_{1}+d_{2}\right)}}\frac{\partial^{4}w}{\partial x^{4}}-\underset{\beta }{\underbrace{\left(d_{3}+d_{4}+d_{5}\right)}}\left(\frac{\partial^{2}w}{\partial x^{2}}\right){\left(\frac{\partial w}{\partial x}\right)}^{2}=0$$

with the dimensionless boundary conditions for the non-contact region

(17) $$w\left(0,t\right)=\frac{\partial w}{\partial x}\left(0,t\right)=\frac{\partial^{2}w}{\partial x^{2}}\left(1,t\right)=0\;\underset{\alpha }{\underbrace{\left(d_{1}+d_{2}\right)}}\frac{\partial^{3}w}{\partial x^{3}}\left(1,t\right)=-\frac{d_{6}}{{\left[\left(\bar{Z}\right)-w\left(1,t\right)-{\bar{d}}_{0}\sin\left(\Omega t\right)\right]}^{2}}$$

## 3. Discretization of the Governing Motion’s Equation

In this section, the governing partial differential equation is discretized by using the Galerkin method. Because the boundary condition is time-dependent, implementing the Galerkin method directly may be difficult. To solve such non-homogeneous boundary conditions, the following process is implemented.

It is assumed that the deflection has the following form [46]

(18) $$w\left(x,t\right)=W\left(x,t\right)+F\left(t\right)\;G\left(x\right)$$

in which $F\left(t\right)$ is obtained from non-homogeneous boundary conditions. $F\left(t\right)$ can be expressed as the following formulation.

(19) $$F\left(t\right)=-\frac{d_{6}}{\alpha {\left[\bar{Z}-w\left(1,t\right)-{\bar{d}}_{0}\sin\left(\Omega t\right)\right]}^{2}}$$

In Equation (18), $G\left(x\right)$ is an arbitrary function satisfying the following conditions:

(20) $$G\left(0\right)=\frac{dG}{dx}\left(0\right)=G\left(1\right)=\frac{dG}{dx}\left(1\right)=\frac{d^{2}G}{dx^{2}}\left(1\right)=0,\;\frac{d^{3}G}{dx^{3}}\left(1\right)=1$$

A suitable assumption for the function $G\left(x\right)$ is chosen as

(21) $$G\left(x\right)=\frac{-1}{6}x^{2}+\frac{1}{2}x^{3}-\frac{1}{2}x^{4}+\frac{1}{6}x^{5}$$

Substituting Equation (18) into Equations (16) and (17), the governing equation is derived as:

(22) $$\begin{array}{ll} \frac{\partial^{2}W}{\partial t^{2}}+c\frac{\partial W}{\partial t} & +\alpha \frac{\partial^{4}W}{\partial x^{4}}-\beta \left(\frac{\partial^{2}W}{\partial x^{2}}\right){\left(\frac{\partial W}{\partial x}\right)}^{2}+G\frac{d^{2}F}{dt^{2}}+cG\frac{dF}{dt}+\alpha F\frac{d^{4}G}{dx^{4}}\\ & -2\beta F\frac{dG}{dx}\;\left(\frac{\partial^{2}W}{\partial x^{2}}\right)\left(\frac{\partial W}{\partial x}\right)-\beta F^{2}{\left(\frac{dG}{dx}\right)}^{2}\left(\frac{\partial^{2}W}{\partial x^{2}}\right)\\ & -\beta F\left(\frac{d^{2}G}{dx}\right){\left(\frac{\partial W}{\partial x}\right)}^{2}-2\beta F^{2}\left(\frac{dG}{dx}\right)\left(\frac{d^{2}G}{dx}\right)\left(\frac{\partial W}{\partial x}\right)\\ & -\beta F^{3}{\left(\frac{dG}{dx}\right)}^{2}\left(\frac{d^{2}G}{dx}\right)={\bar{d}}_{0}{\;\Omega }^{2}\sin\left(\Omega t\right)-c{\bar{d}}_{0}\;\Omega \cos\left(\Omega t\right) \end{array}$$

Also, the corresponding boundary condition for Equation (22) is as follows:

(23) $$W\left(0,t\right)=\;W^{\prime }\left(0,t\right)=\;W^{\prime \prime }\left(1,t\right)=\;W^{\prime \prime \prime }\left(1,t\right)=0$$

The prime notation is derivative with respect to the dimensionless axial coordinate.

It is seen that the non-homogeneous boundary condition has been transformed into a homogeneous one. Now, the Galerkin method is applied to Equations (22) and (23). Since the contribution of the first mode is dominant in comparison to higher modes, the first mode is adopted in the present work.

By applying the variables separation technique, the response of the new variable$\;U\left(x,t\right)$ is supposed to be as:

(24) $$W\left(x,t\right)=q\left(t\right)\phi \left(x\right)$$

in which $q\left(t\right)$ denotes the generalized coordinate, and $\phi \left(x\right)$ stands for the eigenfunction for a clamped-free beam, given by

(25) $$\phi \left(x\right)=\left\{\cos\left(\beta_{1}x\right)-\cos h\left(\beta_{1}x\right)\right\}-\left(\frac{\left\{\cos\left(\beta_{1}\right)+\cos h\left(\beta_{1}\right)\right\}}{\left\{\sin\left(\beta_{1}\right)+\sin h\left(\beta_{1}\right)\right\}}\left\{\sin\left(\beta_{1}x\right)-\sin h\left(\beta_{1}x\right)\right\}\right)$$

where $\beta_{1}=1.8751$.

Substituting Equation (24) into Equation (22) and multiplying the resultant equation by $\phi \left(x\right)$, then integrating with respect to x from 0 to 1, the following governing equation in the time domain is derived as:

(26) $$\ddot{q}+c\dot{q}+\omega_{0}^{2}q-r_{2}q^{3}+r_{3}\ddot{F}+cr_{3}\dot{F}+r_{4}F-r_{5}Fq^{2}-r_{6}F^{2}q-r_{7}Fq^{2}-r_{8}F^{2}q-r_{9}F^{3}=r_{10}{\;\Omega }^{2}\sin\left(\Omega t\right)-cr_{10}\;\Omega \cos\left(\Omega t\right)$$

in which

(27) $$\begin{array}{ll} r_{1}=\int_{0}^{1}\alpha \;\phi \phi^{\prime \prime \prime \prime }dx=\omega_{0}^{2}, & r_{2}=\int_{0}^{1}\beta \phi \phi^{\prime \prime }{\left(\phi^{\prime }\right)}^{2}dx\\ r_{3}=\int_{0}^{1}\phi \;Gdx, & r_{4}=\int_{0}^{1}\alpha \;\phi \;G^{\prime \prime \prime \prime }dx\\ r_{5}=\int_{0}^{1}2\beta \phi G^{\prime }\phi^{\prime }\phi^{\prime \prime }dx, & r_{6}=\int_{0}^{1}\beta \phi G^{\prime }G^{\prime }\phi^{\prime \prime }dx\\ r_{7}=\int_{0}^{1}\beta \phi \phi^{\prime }\phi^{\prime }G^{\prime \prime }dx, & r_{8}=\int_{0}^{1}2\beta \phi \phi^{\prime }G^{\prime }G^{\prime \prime }dx\\ r_{9}=\int_{0}^{1}\beta \phi G^{\prime }G^{\prime }G^{\prime \prime }dx, & r_{10}=\int_{0}^{1}{\bar{d}}_{0}\phi \;dx \end{array}$$

We now simplify the equation of motion by expanding the function $F\left(t\right)$ around zero point, i.e.,

(28) $$F\left(t\right)=k_{1}+k_{2}q\left(t\right)$$

in which

(29) $$k_{1}=-\frac{h_{1}}{{\left(\bar{Z}-g\left(t\right)\right)}^{2}},\;k_{2}=-\frac{h_{2}}{{\left(\bar{Z}-g\left(t\right)\right)}^{3}},\;g\left(t\right)={\bar{d}}_{0}\sin\left(\Omega t\right),\;h_{1}=\frac{d_{6}}{\alpha },\;h_{2}=\frac{2d_{6}\phi \left(1\right)}{\alpha }$$

Substituting Equation (28) into Equation (26), we get

(30) $$M\ddot{q}+C\dot{q}+K_{l}q+K_{q}q^{2}+K_{c}q^{3}=r_{10}{\;\Omega }^{2}\sin\left(\Omega t\right)-cr_{10}\;\Omega \cos\left(\Omega t\right)$$

in which

(31) $$M=1-\frac{h_{2}r_{3}}{{\left(\bar{Z}-g\left(t\right)\right)}^{3}}\;C=c-\frac{c\;h_{2}r_{3}}{{\left(\bar{Z}-g\left(t\right)\right)}^{3}}\;K_{L}=r_{1}+\frac{3h_{1}^{2}h_{2}r_{9}}{{\left(\bar{Z}-g\left(t\right)\right)}^{7}}-\frac{h_{1}^{2}r_{6}}{{\left(\bar{Z}-g\left(t\right)\right)}^{4}}-\frac{h_{1}^{2}r_{8}}{{\left(\bar{Z}-g\left(t\right)\right)}^{4}}-\frac{h_{2}r_{4}}{{\left(\bar{Z}-g\left(t\right)\right)}^{3}}\;K_{q}=\frac{3h_{1}h_{2}^{2}r_{9}}{{\left(\bar{Z}-g\left(t\right)\right)}^{8}}-\frac{2h_{1}h_{2}r_{6}}{{\left(\bar{Z}-g\left(t\right)\right)}^{5}}-\frac{2h_{1}h_{2}r_{8}}{{\left(\bar{Z}-g\left(t\right)\right)}^{5}}+\frac{h_{1}r_{5}}{{\left(\bar{Z}-g\left(t\right)\right)}^{2}}+\frac{h_{1}r_{7}}{{\left(\bar{Z}-g\left(t\right)\right)}^{2}}\;K_{c}=-r_{2}+\frac{h_{2}^{3}r_{9}}{{\left(\bar{Z}-g\left(t\right)\right)}^{9}}-\frac{h_{2}^{2}r_{6}}{{\left(\bar{Z}-g\left(t\right)\right)}^{6}}-\frac{h_{2}^{2}r_{8}}{{\left(\bar{Z}-g\left[t\right]\right)}^{6}}+\frac{h_{2}r_{5}}{{\left(\bar{Z}-g\left[t\right]\right)}^{3}}+\frac{h_{2}r_{7}}{{\left(\bar{Z}-g\left(t\right)\right)}^{3}}$$

To distinguish between parametric and non-parametric resonances, the resultant equation is multiplied by ${\left(\bar{Z}-g\left(t\right)\right)}^{9}$, resulting in

(32) $$\begin{array}{ll} M{\left(\bar{Z}-g\left(t\right)\right)}^{9}\ddot{q} & +C{\left(\bar{Z}-g\left(t\right)\right)}^{9}\dot{q}+K_{l}{\left(\bar{Z}-g\left(t\right)\right)}^{9}q+K_{q}{\left(\bar{Z}-g\left(t\right)\right)}^{9}q^{2}\\ & +K_{c}{\left(\bar{Z}-g\left(t\right)\right)}^{9}q^{3}\\ & =r_{10}{\;\Omega }^{2}\sin\left(\Omega t\right){\left(\bar{Z}-g\left(t\right)\right)}^{9}-cr_{10}\;\Omega \cos\left(\Omega t\right){\left(\bar{Z}-g\left(t\right)\right)}^{9} \end{array}$$

Simplifying the equations by assuming that ${\bar{d}}_{0}$ is too small compared to $\bar{Z}=1$.

(33) $$M_{1}\ddot{q}+C_{1}\dot{q}+K_{L1}q+K_{q1}q^{2}+K_{c1}q^{3}=r_{10}{\;\Omega }^{2}\sin\left(\Omega t\right)-cr_{10}\;\Omega \cos\left(\Omega t\right)$$

in which

(34) $$\begin{array}{l} M_{1}=1-h_{2}r_{3}+\left(6{\bar{d}}_{0}h_{2}r_{3}-9{\bar{d}}_{0}\right)\sin\left(\Omega t\right)\\ C_{1}=c-c\;h_{2}r_{3}+\left(6{\bar{d}}_{0}c\;h_{2}r_{3}-9{\bar{d}}_{0}c\right)\sin\left(\Omega t\right)\\ K_{L1}=3h_{1}^{2}h_{2}r_{9}-h_{1}^{2}r_{6}-h_{1}^{2}r_{8}-h_{2}r_{4}+r_{1}\\ +\left(-6{\bar{d}}_{0}h_{1}^{2}h_{2}r_{9}+5{\bar{d}}_{0}h_{1}^{2}r_{6}+5{\bar{d}}_{0}h_{1}^{2}r_{8}+6{\bar{d}}_{0}h_{2}r_{4}-9{\bar{d}}_{0}r_{1}\right)\sin\left(\Omega t\right)\\ K_{q1}=3h_{1}h_{2}^{2}r_{9}-2h_{1}h_{2}r_{6}-2h_{1}h_{2}r_{8}+h_{1}r_{5}+h_{1}r_{7}\\ +\left(-3{\bar{d}}_{0}h_{1}h_{2}^{2}r_{9}+8{\bar{d}}_{0}h_{1}h_{2}r_{6}+6{\bar{d}}_{0}h_{1}h_{2}r_{8}-7{\bar{d}}_{0}h_{1}r_{5}-7{\bar{d}}_{0}h_{1}r_{7}\right)\sin\left(\Omega t\right)\\ K_{c1}=-r_{2}+h_{2}r_{5}+h_{2}r_{7}-h_{2}^{2}r_{6}-h_{2}^{2}r_{8}+h_{2}^{3}r_{9}\\ +\left(\;3{\bar{d}}_{0}h_{2}^{2}r_{6}+3{\bar{d}}_{0}h_{2}^{2}r_{8}-6{\bar{d}}_{0}h_{2}r_{5}-6{\bar{d}}_{0}h_{2}r_{7}+9{\bar{d}}_{0}r_{2}\right)\sin\left(\Omega t\right) \end{array}$$

## 4. Result and Discussion

In this section, the numerical results for the system under consideration shown in Figure 1 and Figure 2 are illustrated. Unless stated otherwise, the material and geometrical parameters of the AFM cantilever are listed in Table 1. Because the influence of the size effect has been well addressed in previous studies, the material length-scale parameter is taken as zero in the numerical simulation and only has been expressed in the mathematical formulation. The main aim of this section is to investigate the principal parametric resonance of the system [47].

**Figure 3:**
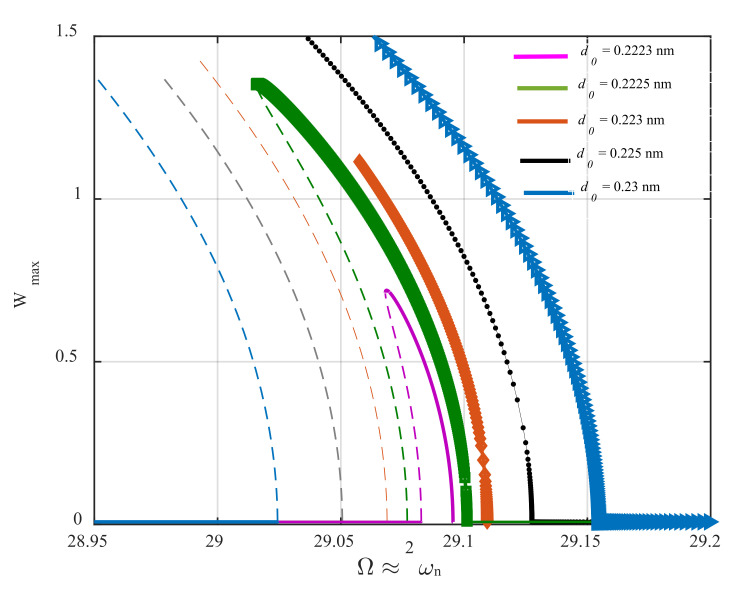
Frequency-response behavior of the AFM in the neighbor of its principal parametric resonance for different values of the base-excitation amplitude.

As seen in Equations (33) and (34), the combination of van der Waals force and base-excitation results in time-dependent linear inertia, stiffness, damping terms, and harmonically time-varying nonlinear stiffness terms, creating parametric parameters excitations. Moreover, the AFM is affected by two external excitations having the same frequencies, which are equal to the frequency of the base excitation. It is observed that the frequency of all parametric excitations appearing in the linear, quadratic, and cubic nonlinearities is equal to that of the external excitation. In this work, the quality factor of the system is assumed to be 200, defining a low-damped vibrating system. Under this condition, if the frequency of the parametric excitation varies twice the fundamental natural frequency of the AFM, principal parametric resonance is activated for small values of the base-excitation amplitude, $d_{0}$. In other words, for the quality factor of 200, the activation level of parametric resonance is reached at small values of $d_{0}$ that can let us neglect the contribution of the time-varying term existing in the inertia term. Another point that is worth mentioning is that, while the frequency of the base excitation is close to two times the first natural frequency, the sub-harmonic resonance of the first mode can be activated by the external excitation; however, its activation level is much greater than that of the principal parametric resonance. Therefore, it can be concluded that this type of nonlinear resonance does not contribute to the system’s response within the variation range of $d_{0}$, which results in parametric resonance. With this in mind, the frequency-response behavior of the AFM is captured for different values of the base excitation amplitude and illustrated in Figure 3.

The figure shows that the frequency–response curves are composed of trivial stable, trivial unstable, nontrivial stable, and nontrivial unstable branches. The term *trivial* refers to the periodic response with zero amplitude; however, *nontrivial* phrase returns to the periodic orbit with non-zero amplitude. Moreover, the term *stable* clarifies that the system’s states are absorbed by the periodic orbit after the system is disturbed; however, this is not the case for the *unstable* term, meaning that any small disturbance applied to the system’s dynamics causes the system’s states to get off the periodic orbit without the ability of returning back to it. The system’s dynamics begin with stable zero-amplitude solutions and continue until primary Hopf bifurcation (sub-critical) occurs, and the stable branch loses its stability. Further increasing the parametric frequency, the unstable trivial solutions retrieve their stability at super-critical Hopf bifurcation points. In addition, for the smallest value of $d_{0}$ parameter close to the activation level, the stable and unstable nontrivial branches meet at the cyclic-fold bifurcation point at Ω≈29.07; however, this is not the case for larger values of the parameter. As the parametric pump enhances, the resonance bandwidth becomes broader. The non-zero stable and unstable solutions meet at a displacement value beyond the gap distance between the microbeam and the substrate. Furthermore, it can be inferred that the quadratic and cubic nonlinearities arising from the intermolecular force induce a softening effect on the steady-state dynamics of the AFM, making the nontrivial solution branches bend to the left. It should be mentioned that $W_{\max}$ is the displacement of the cantilever tip. For better describing the frequency–response curves, it should be noted that there has been considered a set of two curves corresponding to each color. For instance, the blue plot drawn in Figure 3 is composed of two curves; the one which is marked by a star showing the stable solution, and the one that is mark-free shows the unstable solution branch corresponding to the same value of $d_{0}$.

**Figure 4:**
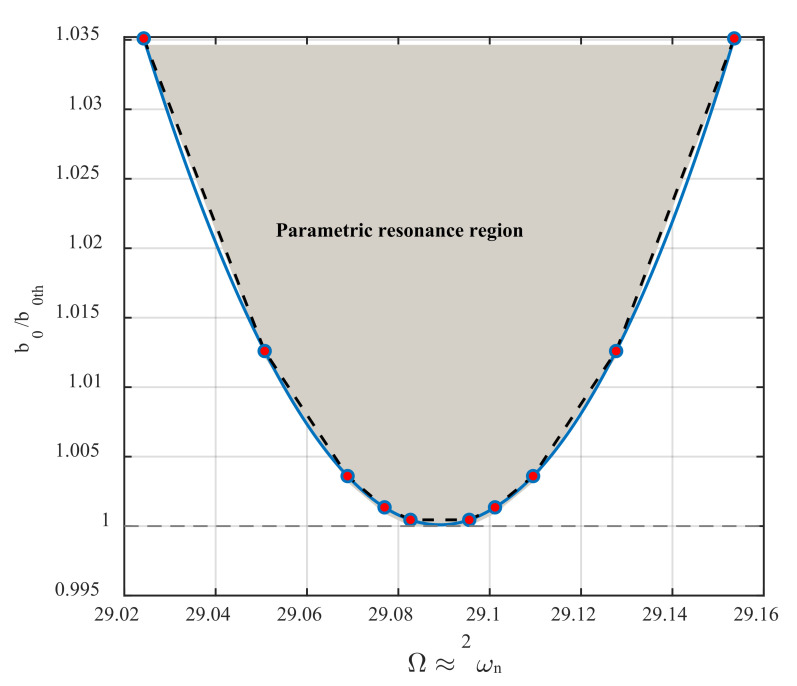
Instability tongue. The loci of both sub- and super-critical primary Hopf bifurcation points define the boundary of the parametric resonance region, where the threshold value for the base excitation amplitude is $d_{th}=0.222\;\mathrm{nm}$.

Figure 4 demonstrates the loci of the primary Hopf bifurcation points for different values of the excitation amplitude. The vertical axis shows the ratio between the excitation amplitude to its threshold, leading to parametric resonance activation. As seen in the figure, while this ratio is close to one from below, there is still no bifurcation in the system’s dynamics. However, while this ratio is close to one from above, two bifurcation points are occurring at two frequencies so close to each other that the left ones return to the sub-critical, and the right ones introduce the position of the super-critical Hopf bifurcation points. As the amplitude of the parametric excitation grows, the distance between these two points increases. The grey area bounded by the fitted blue curve displays the parametric resonance region, known as the instability tongue. It is worth noting that if the parametric excitations are not accompanied by external excitation, the parametric noise squeezing effect is potent to happen while the excitation amplitude is below the activation level. However, this phenomenon cannot occur in the present dynamics, because the parametric excitation is collaborated by external excitation with the same excitation frequency. Inspecting the numerical values on the vertical axis, one can find that the parametric resonance bandwidth is extremely sensitive to the excitation amplitude.

**Figure 5:**
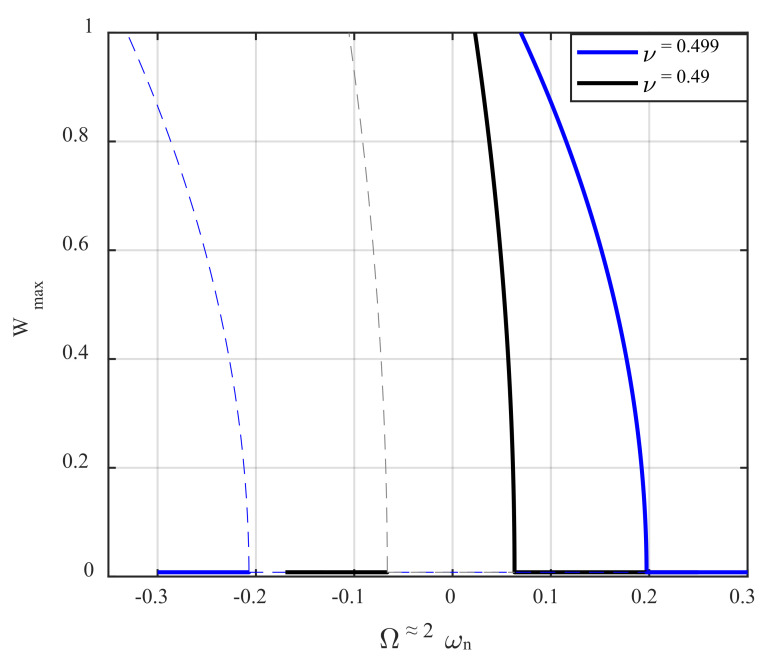
The impact of Poisson’s ratio on the frequency-displacement behavior of the AFM, for $d_{0}=0.23\;\mathrm{nm}$. The dashed and solid lines are unstable and stable branches, respectively.

Figure 5 illustrates the influence of the incompressibility (ν) on the frequency-response behavior of the AFM system by choosing two different values for Poisson’s ratio, for $d_{0}=0.23$ nm. Increasing the value of Poisson’s ratio causes the structure to become stiffer and more rigid, and therefore, its natural frequencies grow remarkably. With this in mind, the frequency of the parametric excitation is swept in the neighbour of twice the first natural frequency, which is evaluated for that specific Poisson’s ratio. In order to have a precise evaluation of how the impact of Poisson’s ratio influences the slope of the frequency–response curve, the displacement amplitude is plotted versus the difference between the excitation frequency and two times the natural frequency. It is found from the figure that, not only the resonance region becomes more expansive, but also the softening trend is intensified. At the same time, the resonator is made from a more incompressible material.

### Profile Height Detection Mechanism

**Figure 6:**
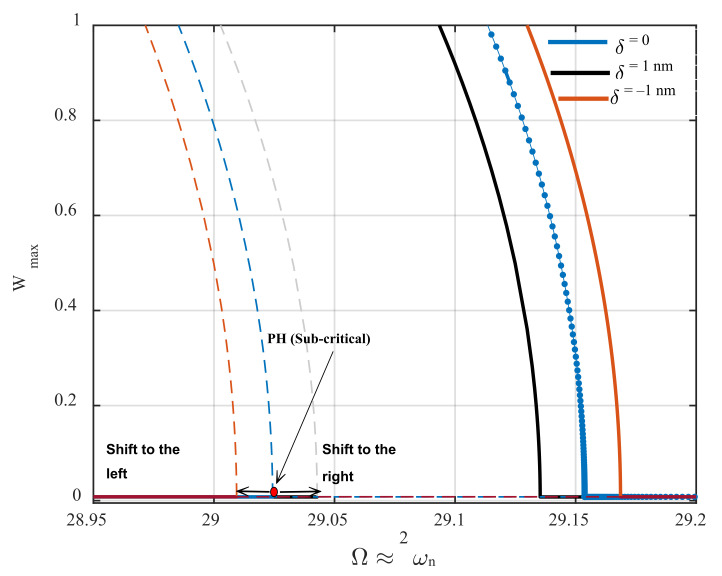
The influence of the surface profile variations on the primary Hopf bifurcation points near parametric resonance for the base excitation amplitude of 0.23 nm. The dashed and solid lines are unstable and stable branches, respectively.

In this section, a sensing mechanism to detect the height of a sample’s profile that is implemented based on the principal parametric resonance characteristics is proposed. First, it is shown that while the AFM is operating near its parametric resonance, the bifurcation points’ position is highly affected by the gap distance between the microcantilever and its sample underneath. Figure 6 demonstrates the frequency-response behavior of the AFM for the excitation amplitude of $d_{0}=0.23\;\mathrm{nm}$. Here, the initial gap distance is set to be 60 nm. As seen in the figure, the sub-critical and super-critical Hopf bifurcation points shift to the left and to the right, respectively, while there is a 1 nm rise in the height of the sample profile (orange curve). An increment in the height of the sample profile causes the gap distance between the cantilever tip and the sample top surface to decrease. Therefore, the impact of van der Waals’s force enhances, and consequently, the activation level decreases so that the resonance region becomes broader while the excitation amplitude is kept constant. This 1 nm rise in the height of the sample causes a 0.01 nondimensional frequency shift at primary Hopf bifurcation points. On the other hand, if the sample profile undergoes a 1 nm surface depression, extending the gap between the cantilever tip and the top surface of the sample, weakening the van der Waals force, the activation level of the parametric resonance increases. Hence, the resonance region shrinks while the parametric pump is kept constant, meaning the sub-critical and super-critical Hopf bifurcation points shift to the right and the left, respectively (black curve). As observed in the figure, the frequency shift caused by the rise in the height of the sample profile is quite a bit smaller than that caused by surface depression. Here, the frequency shift at bifurcation points caused by a 1 nm profile depression is obtained at about 0.0125. In Figure 6, PH stands for primary Hopf bifurcation.

**Figure 7:**
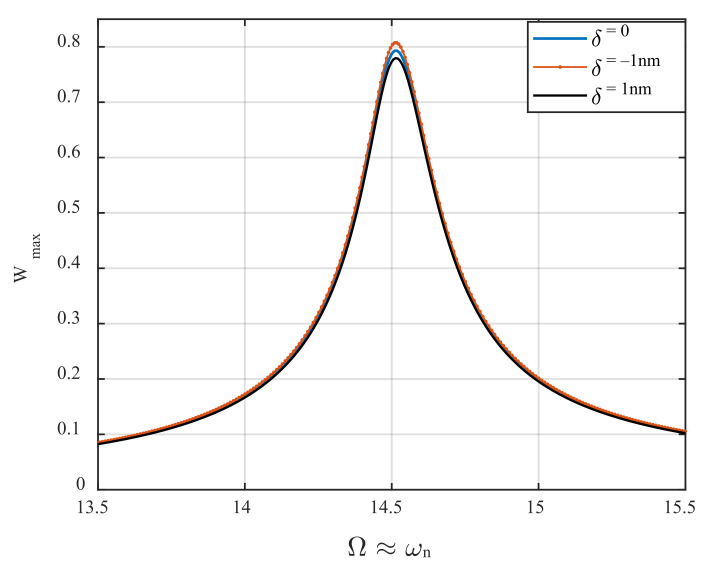
The influence of the surface profile variations on the resonance amplitude of the microcantilever near primary resonance, for $d_{0}=0.15\;\mathrm{nm}$.

The frequency-response behavior of the AFM device near the primary resonance of its first mode is illustrated in Figure 7. To monitor the steady-state dynamic response of the system near its primary resonance, the external excitation frequency is set to be changing in the vicinity of the first natural frequency, and the displacement amplitude of the periodic orbit is recorded for each specific value of the excitation frequency. As seen in the figure, the frequency-displacement behavior does not experience bifurcation for the excitation amplitude of $d_{0}=0.15\;\mathrm{nm}$; however, cyclic-fold bifurcation occurs beyond the gap distance for larger values of the base excitation amplitude, which is not meaningful, and we prevented presenting that result here. It is worth mentioning that for this case, the secondary parametric resonance of the first mode $\left(\Omega \approx \omega_{n}\right)$ of oscillation is probable to happen because, as stated before, the frequency of the parametric terms is identical to that of the external stimulus. While the excitation frequency varies near the natural frequency of the first mode itself, the system has the potential to experience the combination of both primary resonance (due to external/direct excitation) and secondary parametric resonance (due to parametric excitation) of the first mode. However, the secondary parametric resonance $\left(\Omega \approx \omega_{n}\right)$ requires a higher level of activation compared to the primary parametric resonance $\left(\Omega \approx 2\omega_{n}\right)$. Therefore, it cannot be motivated for the proposed AFM, because the cantilever experiences tapping mode, which is caused by large-amplitude oscillation due to direct excitation. Inspecting the figure, it is seen that while there is a 1 nm increase in the height of the sample profile, the resonance amplitude grows slightly (orange curve) compared to the case in which there is no variation in the surface height (blue curve). On the other hand, the displacement amplitude at resonance drops quite a bit while the surface of the sample undergoes a 1 nm depression. Conversely, these variations in the profile configuration do not result in a change in the primary resonance frequency. In this work, the authors propose an effective detection mechanism based on amplitude shift at bifurcation points that are highly sensitive to detecting the high variations of samples’ surface profiles. The AFM needs to be run near its parametric resonance zone instead of the primary resonance region.

**Figure 8:**
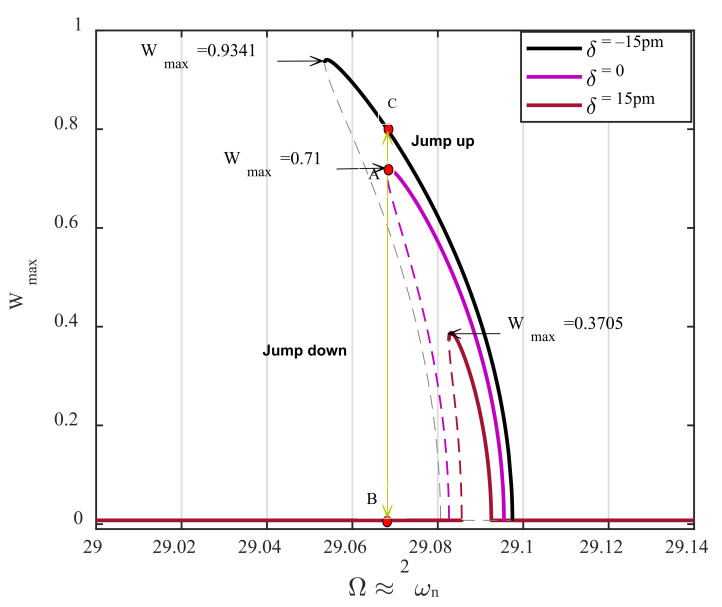
The influence of the surface profile variations on the amplitude of the resonator’s displacement at cyclic-fold bifurcation near parametric resonance, for $\frac{d_{0}}{d_{th}}=1.001$. The dashed and solid lines are unstable and stable branches, respectively.

Figure 8 depicts the frequency-displacement amplitude of the AFM near its parametric resonance for a parametric pump which is slightly above the activation level, $\frac{d_{0}}{d_{th}}=1.001$. For the case in which there is neither bulge nor surface depression on the surface profile (violet curve), a cyclic-fold bifurcation exists at Ω=29.068. This dynamic behavior corresponds to the case where the cantilever tip is precisely on top of the sample area from which the initial gap distance, $\hat{Z}$, is measured. This profile status is considered the surface baseline, provided that any variation in the height of the sample profile concerning this baseline is denoted by δ. As observed in the figure, the frequency–response curve shifts to the right once the distance between the microcantilever tip and the surface profile increases by 15 pm; namely, the AFM faces a surface depression of 15 pm on the sample profile. Because the AFM is already set to be operating at point A, this right shift in the steady-state behavior of the device causes a significant drop in the displacement amplitude, jumping from point A down to point B with zero amplitude. The AFM’s sensitivity can be obtained by evaluating the dimensional displacement drop to the change in the profile height. Accordingly, the sensitivity of the proposed method is as follows:

(35) $$S_{D}{}_{\delta }^{w}=\frac{\Delta w}{\Delta \delta }=\frac{0.71\times 60\;\mathrm{nm}}{15\;\mathrm{pm}}=2840\frac{\mathrm{amplitude}\;\left(\mathrm{nm}\right)}{\delta \;\left(\mathrm{nm}\right)}$$

On the other hand, the frequency–response curve shifts to the left while there is a 15 pm rise in the sample profile, so the resonator’s response experiences a jump up to point C. Although this enhancement in the displacement amplitude is smaller than what is achieved for surface depression, the detection sensitivity is still considerable compared to the case where the cantilever is actuated near its primary resonance (comparing Figure 7 and Figure 8). Similarly, for the case in which the distance between the AFM tip and the sample profile diminishes, the sensitivity of the device is obtained as follows,

(36) $$S_{R}{}_{\delta }^{w}=\frac{\Delta w}{\Delta \delta }=\frac{\left(0.8-0.71\right)\times 60\;\mathrm{nm}}{15\;\mathrm{pm}}=360\frac{\mathrm{amplitude}\;\left(\mathrm{nm}\right)}{\delta \;\left(\mathrm{nm}\right)}$$

Scientifically speaking, because of the softening behavior of the AFM, the response amplitude drops from the stable nontrivial branch down to the stable trivial branch. At the same time, there is a positive variation in the height of the sample (surface depression), leading to ultrahigh sensitivity. However, this is not the case while there are negative variations in the height of the sample profile, meaning that the system’s response jumps from a stable nontrivial branch up to a new generated stable nontrivial solution branch, degrading the AFM sensitivity. This can be improved by designing a tuneable AFM that can show both softening and hardening behavior near its parametric resonance regime. It is worth noting that the most challenging part in achieving ultrahigh sensitivity in AFMs returns to the employment of the base excitation amplitude. The finer the resolution of the parametric pump (parametric excitation amplitude), the higher the possibility for the implementation of the proposed detection mechanism. This means that the suggested method requires the capability of changing the base excitation amplitude with fine resolution. The proposed method can be feasibly employed once this concern is responded to.

In this paper, a micropolar model Equation (4) suitable for hyperelastic materials was utilized. However, other hyperelastic models can be utilized for hyperelastic microcantilevers; for example, the Gent model [48], generalized neo-Hookean models [49], Gent-Gent model [50], etc.

The foremost aspect of the analysis in the present work is that the system operates in parametric resonance regime. We have identified such a regime for non-contact AFM that is common in many real setups. For other kinds of AFM, it is essential to seek the presence of parametric resonance in the system first. Then, the analysis of the current study can be developed for the system.

## 5. Conclusions

This work examined the nonlinear resonance of a hyperelastic-based cantilever AFM in the non-contact region so that the non-contact force is modeled using the van der Waals force. A vertical base excitation is used to vibrate the AFM. A hyperelastic model combined with the modified couple stress theory is proposed to incorporate the elastic energy stored in the microcantilever caused by deformation. The obtained equation of motion is discretized via a developed Galerkin method. Furthermore, an efficient detection mechanism based on principal parametric resonance, assessing surface depression and height increase of a sample profile, is proposed. The following concluding remarks can be deduced from the presented numerical results.

- The hyperelastic microcantilever of the AFM device undergoes softening behavior near its principal parametric resonance.
- The frequency–displacement curve governing the resonator’s dynamics comprises stable trivial, unstable trivial, stable nontrivial, and unstable nontrivial branches.
- The resonance of the AFM exhibits both super- and sub-critical Hopf bifurcations for the considerable value of $d_{0}$, and cyclic-fold bifurcation, for a small value of $d_{0}$.
- Increasing the incompressibility condition (higher values of the Poisson’s ratio) results in stronger softening nonlinearity, and the resonance bandwidth becomes wider.
- Surface profile depression and rise in the height of a surface profile can be detected by inspecting the bifurcation points’ position.
- According to the sensitivity analysis presented in Equation (35), the proposed AFM can detect surface depression in the order of a picometer, providing ultrahigh sensitivity.

## Figures and Tables

**Table 1:** The material and geometrical parameters of the AFM cantilever.

Parameter	Value
Modulus of elasticity, E	3 GPa
Length, L	225 μm
Cross-section area, A	$7.02\times 10^{-11}{\;m}^{2}$
The second moment of area, I	$3.57\times 10^{-23}{\;m}^{4}$
Hamaker constant, H	$2.96\times 10^{-19}$ J
Tip radius, R	10 nm
Initial tip–sample distance, $\hat{Z}$	60 nm
Poisson’s ratio ν	0.49

## Data Availability

There is no data for sharing and all data are available within the paper.
